# Autoimmune cholangitis mimicking a klatskin tumor: a case report

**DOI:** 10.1186/1752-1947-5-485

**Published:** 2011-09-28

**Authors:** Alexandra Shingina, David Owen, Charles Zwirewich, Baljinder Salh

**Affiliations:** 1 Department of Internal Medicine, Vancouver General Hospital, 855 West 12th Avenue, Vancouver, V5Z 1N1, Canada; 2 Department of Anatomical Pathology, Vancouver General Hospital, 855 West 12th Avenue, Vancouver, V5Z 1N1, Canada; 3 Department of Gastrointestinal Radiology, Vancouver General Hospital, 855 West 12th Avenue, Vancouver, V5Z 1N1, Canada; 4 Division of Gastroenterology, 2775, Laurel Street, Vancouver, British Columbia, V5Z 1M9, Canada

## Abstract

**Introduction:**

Autoimmune cholangitis remains an elusive manifestation of immunoglobulin G4-associated systemic disease most commonly encountered in patients with autoimmune pancreatitis. No strict diagnostic criteria have been described to date and diagnosis mainly relies on a combination of clinical and histopathologic findings. It is hence even more challenging to diagnose autoimmune cholangitis in patients with late or atypical presentations, such as without concomitant pancreatic involvement. Early diagnosis of this rare disorder can significantly improve outcomes considering high rates of surgical intervention, as well as high relapse rates in the absence of steroid treatment. To the best of our knowledge the literature is quite sparse on cases with atypical presentations of autoimmune cholangitis.

**Case presentation:**

We report a case of a previously healthy 65-year-old man of Middle-Eastern origin, with a history of pancreatic insufficiency of unknown etiology, evaluated for elevated liver function tests found incidentally on a routine physical examination. Imaging studies revealed an atrophic pancreas and biliary duct dilatation consistent with obstruction. Subsequent endoscopic retrograde cholangiopancreatography showed a bile duct narrowing pattern suggestive of cholangiocarcinoma, but brushings failed to reveal malignant cells. Our patient proceeded to undergo surgical resection. Histological examination of the resected mass revealed lymphoplasmacytic infiltrate with no malignant features. Our patient returned three months later with persistently high liver function tests and no evidence of biliary obstruction on imaging. A presumptive diagnosis of autoimmune cholangitis was made and our patient's symptoms resolved after a short course of an oral steroid regimen. Post factum staining of the resection specimen revealed an immunoglobulin G4 antibody positive immune cell infiltrate, consistent with the proposed diagnosis.

**Conclusion:**

Our case thus highlights the importance of clinician awareness of the autoimmune spectrum of biliary pathologies when confronted with atypical clinical presentations, the paucity of diagnostic measures and the benefit from long-term steroid and/or immunosuppressive treatment.

## Introduction

Autoimmune pancreatitis (AIP) was initially described by Yoshida and colleagues in 1995 to represent a type of chronic pancreatitis with characteristic histopathologic and imaging findings [1]. Based on clinical and laboratory features, two types of AIP are described in the literature. Type I AIP is thought be a pancreatic manifestation of immunoglobulin (Ig) G4-associated systemic disease characterized by lymphoplasmacytic infiltrate and serum elevation of IgG4 levels [2]. Type II AIP shows less correlation with high IgG4 levels, occurring in a younger population and typically characterized by granulocytic epithelial lesions [3].

Although several diagnostic criteria have been proposed for type I AIP such as HISORt [4] and the Asian Consensus Criteria [5], type II AIP remains largely a clinical diagnosis. Interestingly, it was reported that only 44% of patients with both types of AIP will have elevated serum IgG4 levels, rendering the diagnosis in the remainder of the group more challenging [6].

The importance of recognizing the possibility of AIP-associated disease is particularly important in patients with atypical as well as non-pancreatic presentation, or those caught in later stages of the disease. Prompt initiation of steroid treatment in these individuals could greatly improve outcomes, at least in part, by the avoidance of surgery. We would thus like to share our experience in encountering a case of serum IgG4-negative, steroid-responsive sclerosing cholangitis in a patient with pancreatic insufficiency.

## Case presentation

A previously healthy 65-year-old man of Middle-Eastern origin, with a past medical history of hypertension, was initially referred to our gastroenterology service for evaluation of a suspected gastrointestinal bleed. Upper and lower endoscopies failed to reveal an active source of bleeding at that time, however he did have *Helicobacter pylori*-associated gastritis, which was treated. On a follow-up appointment our patient described a history of diarrhea with passage of greasy stool of several months duration associated with 13.6 kg weight loss but no associated abdominal pain. On further questioning, our patient described a history of binge alcohol consumption over five years prior to presentation. His physical exam was unremarkable and laboratory investigations revealed mild anemia, with hemoglobin (Hg) 127 g/L, normal liver function tests (LFTs), negative stool cultures, increased fecal fat of 72 g/day, fecal elastase below 15 ug/g of stool, and elevated blood sugar. Ultrasound investigation of his abdomen was unremarkable and computed tomography (CT) of his abdomen revealed an atrophic pancreas with no focal lesions. Our patient was presumed to have chronic alcoholic pancreatitis, although the absence of abdominal pain was puzzling, and was prescribed pancreatic enzyme replacement therapy with significant clinical improvement.

**Figure 1:**
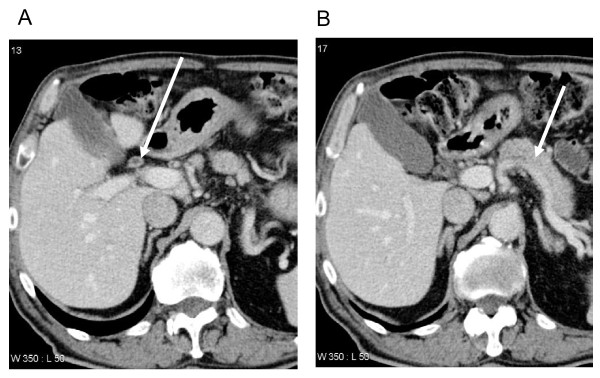
**Abdominal CT scan**. **(A) **Arrow pointing to bile duct wall thickening. **(B) **Arrow outlining atrophic pancreas.

**Figure 2:**
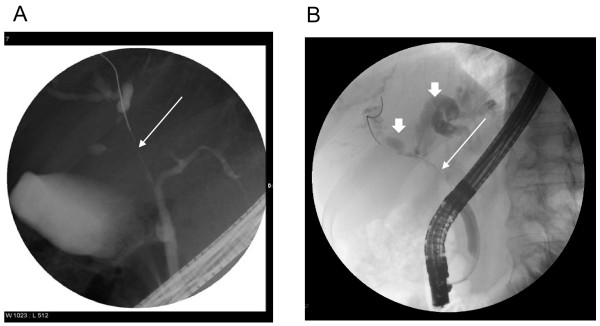
**ERCP findings**. **(A) **Stenotic region within right intrahepatic biliary tree. **(B) **Right and left hepatic ductal dilation (short arrows) above a typical hilar (Klatskin tumor, long arrow) stricture. A guidewire has been passed across the stricture into the right hepatic duct.

One year later our patient was referred again for an evaluation of malaise and elevated LFTs; again there was no complaint of abdominal pain. His alkaline phosphatase (ALP) level was found to be 265 U/L, gamma- glutamyltransferase (γ-GT) 236 U/L, alanine transaminase (ALT) 138 U/L and aspartate transaminase (AST) 94 U/L in the setting of normal lipase and bilirubin levels. Our patient was not jaundiced on exam; carbohydrate antigen (CA) 19-9 was elevated at 210 U/ml with a normal carcinoembryonic antigen (CEA) level. Abdominal ultrasound revealed right-sided intrahepatic bile duct dilatation suggestive of an obstruction. A subsequent abdominal CT scan showed an irregular soft tissue mass in his right distal hepatic duct proximal to the confluence (Figure 1A), and a somewhat atrophic pancreas (Figure 1B). Endoscopic retrograde cholangiopancreatography (ERCP) shortly after identified a long stenotic region within the right intrahepatic biliary tree with mild intermittent left-sided dilatation (Figure 2A). The overall picture was consistent with a presentation of Klatskin tumor (a representative picture of which, from an unrelated patient, is shown in Figure 2B), given the elevated CA19-9 level. Our patient then underwent bile duct resection, cholecystectomy and extended right hepatectomy with Roux-en-Y reconstruction. Intraoperatively, a firm mass was palpated at the hilum of his liver extending into the right ductal system. Our patient had an uneventful postoperative recovery with complete normalization of liver enzymes at the time of discharge.

**Figure 3:**
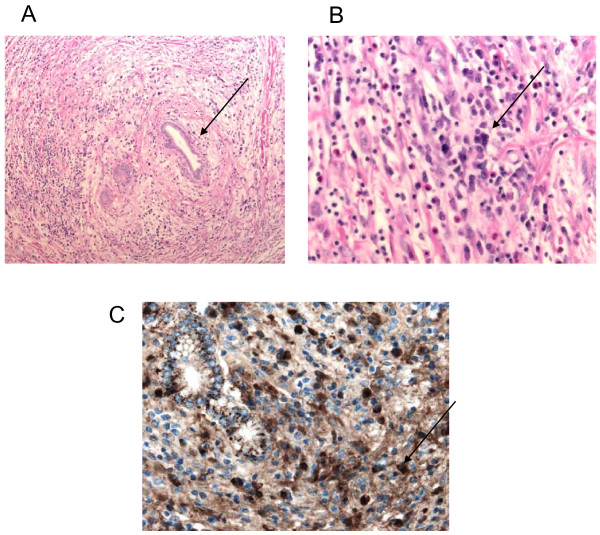
**Histopathology of the resected mass**. **(A) **Hematoxylin and eosin staining showing thickened bile duct. **(B) **Hematoxylin and eosin staining revealing lymphoplasmacytic infiltrate, arrow pointing to plasma cell. **(C) **IgG4 antibody staining revealing more than 55 IgG4 positive cells per high power field.

Subsequent results of the pathology revealed a fibroinflammatory rather than malignant nature of the resected mass, with predominance of a lymphoplasmacytic infiltrate (Figure 3A, B). The liver parenchyma was normal and bile ducts showed proliferation and fibrosis as well as moderate chronic inflammatory infiltrate. Post factum histological staining of the specimen revealed a large IgG4 antibody positive infiltrate, with 55 positive cells per high power field (Figure 3C). Additional serological tests were ordered during a postoperative patient follow-up appointment. These were negative for serum antinuclear antibody and rheumatoid factor, as well as showing hypergammaglobulinemia with a surprisingly low IgG4 level.

The patient returned three months after surgery with malaise, fatigue and a reduced appetite. His laboratory studies revealed abnormal liver enzymes: total/direct bilirubin 21/9 umol/L, ALP 821 U/L, γ-GT 624 U/L, ALT 127 U/L and AST 143 U/L. Abdominal ultrasound revealed the normal appearance of the residual liver with no biliary tract dilatation. Given the histology and positive immunochemical staining for IgG4 positive cells, our patient was then prescribed a trial of a steroid regimen, which led to complete normalization of liver enzymes within three months. After tapering of the steroid therapy three months later our patient developed significant submandibular gland swelling, which again responded to re-introduction of corticosteroids. He now remains well on maintenance azathioprine treatment, 15 months after presenting with unilateral bile duct obstruction.

## Discussion

Autoimmune cholangitis (AIC) is a term that has recently been coined to describe a sclerosing cholangitis associated with IgG4-associated systemic disease. Thus far no diagnostic criteria have been proposed and it remains largely a clinical diagnosis based on histopathological and laboratory parameters. The occasional absence of pancreatic involvement in these patients has been previously reported [7,8].

Despite the rarity of AIC, it remains crucial to be alert to its possibility, especially in patients with cholangitis and underlying unexplained pancreatic disease. We have described a case of a serum IgG4-negative patient who underwent laparotomy for a suspected Klatskin tumor that revealed a lymphocytoplasmic infiltrate staining heavily for IgG4. A previous diagnosis of both endocrine and exocrine pancreatic insufficiency in this patient, with a remote binge drinking history and no history of abdominal pain, is worth re-examining and possibly attributing to the late stage of autoimmune pancreatitis. The remarkable response to steroids further supports an autoimmune origin.

The natural course of AIP-associated cholangitis involves relapses. An examination of a series of 53 AIC patients with a mean follow-up of 38 months demonstrated relapse of 44% and 53% postoperative and post-steroid-withdrawal respectively [9]. Similar observations have been reported in patients with AIP. Kamisawa and colleagues reviewed 563 patients with AIP and showed a statistically significant decrease in the relapse rate of patients treated with maintenance low-dose steroid therapy [10]. It is thus essential to initiate steroid treatment as soon as the suspicion of an autoimmune component is strong enough, while awaiting definitive testing.

We have then reviewed the literature in an attempt to identify what could have aided in the early diagnosis of this patient and could potentially reduce morbidity associated with unnecessary surgical treatment.

The difficulty in preoperative diagnosis was explored by Itoh *et al.*, where radiographic findings in 15 patients with a postoperative diagnosis of AIP were reviewed and failed to demonstrate specific nuances that would differentiate AIC from other biliary disease [11]. This suggests that imaging studies, although essential in investigations of patient symptoms, might not be helpful in detecting the precise etiology of strictures.

Measurement of serum IgG4 levels is not currently recommended as routine without a high index of suspicion for autoimmune origin in patients with gastrointestinal complaints. In addition, as mentioned previously, only 44% of patients with IgG4-associated disease confirmed by biopsy had elevated levels of serum IgG4, rendering this test of equivocal value.

We then hypothesized that perhaps steroid treatment should have been initiated right after the histology of the resected mass described a lymphocytoplasmic infiltrate. We searched the literature for similar cases, and found several cases of patients who have undergone laparotomies for suspected malignant bile duct strictures with postoperative histopathological findings suggestive of AIC [12,13]. In a recent case series, Erdogan and colleagues described 185 patients with benign bile duct strictures who have undergone laparotomy for suspected malignant disease; 15 of these had lymphocytoplasmic infiltrate with only two staining positive for IgG4 [14]. This suggests that histopathology alone is insufficient for the diagnosis and could result in unjustifiably subjecting patients to steroid regimen side effects. IgG4 antibody staining, however, seems to be the only test that allows the diagnosis to be established definitively.

## Conclusion

Our case report highlights the importance of clinician awareness of the possibility of an autoimmune origin of pancreatitis and cholangitis, especially in patients presenting with atypical or late presentations. The challenge still lies in the lack of early diagnostic tests to evaluate biliary strictures that could prevent significant surgical interventions in these patients. Routine IgG4 staining on benign lesions obtained at biopsy in patients with obstructive cholangitis could potentially aid in early initiation of steroid treatment.

## Consent

Written informed consent was obtained from the patient for publication of this case report and any accompanying images. A copy of the written consent is available for review by the Editor-in-Chief of this journal.

## Competing interests

The authors declare that they have no competing interests.

## Authors' contributions

BS provided patient information and was a major contributor in writing and editing the manuscript. AS collected data and composed the preliminary manuscript. DO performed the histological examination of the specimen. CZ provided the CT scan and ERCP images. All authors read and approved the final manuscript.
